# Sweet taste of heavy water

**DOI:** 10.1038/s42003-021-01964-y

**Published:** 2021-04-06

**Authors:** Natalie Ben Abu, Philip E. Mason, Hadar Klein, Nitzan Dubovski, Yaron Ben Shoshan-Galeczki, Einav Malach, Veronika Pražienková, Lenka Maletínská, Carmelo Tempra, Victor Cruces Chamorro, Josef Cvačka, Maik Behrens, Masha Y. Niv, Pavel Jungwirth

**Affiliations:** 1 The Institute of Biochemistry, Food Science and Nutrition, The Robert H Smith Faculty of Agriculture, Food and Environment, The Hebrew University of Jerusalem, Rehovot, Israel; 2 The Fritz Habe Center for Molecular Dynamics, The Hebrew University of Jerusalem, Jerusalem, Israel; 3 Institute of Organic Chemistry and Biochemistry, Czech Academy of Sciences, Prague 6, Czech Republic; 4 Leibniz-Institute for Food Systems Biology at the Technical University of Munich, Freising, Germany

**Keywords:** Biophysical chemistry, Computational biophysics

## Abstract

Hydrogen to deuterium isotopic substitution has only a minor effect on physical and chemical properties of water and, as such, is not supposed to influence its neutral taste. Here we conclusively demonstrate that humans are, nevertheless, able to distinguish D_2_O from H_2_O by taste. Indeed, highly purified heavy water has a distinctly sweeter taste than same-purity normal water and can add to perceived sweetness of sweeteners. In contrast, mice do not prefer D_2_O over H_2_O, indicating that they are not likely to perceive heavy water as sweet. HEK 293T cells transfected with the TAS1R2/TAS1R3 heterodimer and chimeric G-proteins are activated by D_2_O but not by H_2_O. Lactisole, which is a known sweetness inhibitor acting via the TAS1R3 monomer of the TAS1R2/TAS1R3, suppresses the sweetness of D_2_O in human sensory tests, as well as the calcium release elicited by D_2_O in sweet taste receptor-expressing cells. The present multifaceted experimental study, complemented by homology modelling and molecular dynamics simulations, resolves a long-standing controversy about the taste of heavy water, shows that its sweet taste is mediated by the human TAS1R2/TAS1R3 taste receptor, and opens way to future studies of the detailed mechanism of action.

## Abstract (web summary)

Ben Abu, Mason and colleagues use molecular dynamics, cell-based experiments, mouse models, and human subjects to determine that, unlike ordinary water, heavy water tastes sweet to humans, but not mice. Mechanistically, this effect is mediated by the human TAS1R/TAS1R3 sweet taste receptor.

## Introduction

Heavy water, D_2_O, has fascinated researchers since the discovery of deuterium by Urey in 1931^1,2^. The most notable difference in physical properties between D_2_O and H_2_O is the roughly 10% higher density of the former liquid, which is mostly a trivial consequence of deuterium being about twice as heavy as hydrogen. A more subtle effect of deuteration is the formation of slightly stronger hydrogen (or deuterium) bonds in D_2_O as compared to H_2_O^3,4^. This results in a small increase of the freezing and boiling points by 3.8 °C and 1.4 °C, respectively, and in a slight increase of 0.44 in pH (or pD) of pure water upon deuteration^5^. In comparison, a mere dissolution of atmospheric CO_2_ and subsequent formation of dilute carbonic acid in open containers has a significantly stronger influence on the pH of water, changing it by more than one unit^6^.

Biological effects are observable for high doses of D_2_O. While bacteria or yeasts can function in practically pure D_2_O, albeit with somewhat hindered growth rate^7–9^, for higher organisms damaging effects on cell division and general metabolism occur at around 25% deuteration, with lethal conditions for plants and animals typically occurring at ~40–50% deuteration of the body water^2,10,11^. Small levels of deuteration are, nevertheless, harmless. This is understandable given the fact that about 1 in every 6400 hydrogens in nature is found in its stable isotope form of deuterium^12^. Oral doses of several milliliters of D_2_O are safe for humans^13^ and are used in the isotopic form D_2_^18^O for metabolic measurements in clinical praxis (known as “doubly labeled water” technique)^14^. Probably the best-established effect of D_2_O is the increase of the circadian oscillation length upon its administration to both animals and plants. This has been attributed to a general slowdown of metabolism upon deuteration, although the exact mechanism of this effect is unknown^15,16^.

A long-standing unresolved puzzle concerns the taste of heavy water. There is anecdotal evidence from the 1930s that the taste of pure D_2_O is distinct from the neutral one of pure H_2_O, being described mostly as “sweet”^17^. However, Urey and Failla addressed this question in 1935 concluding authoritatively that upon tasting “neither of us could detect the slightest difference between the taste of ordinary distilled water and the taste of pure heavy water”^18^. This had, with a rare exception^19^, an inhibitive effect on further human studies, with research concerning effects of D_2_O focusing primarily on animal or cell models. Experiments in animals indicated that rats developed aversion toward D_2_O when deuteration of their body water reached harmful levels, but there is conflicting evidence regarding their ability to taste heavy water or use other cues to avoid it^20,21^.

**Fig. 1:**
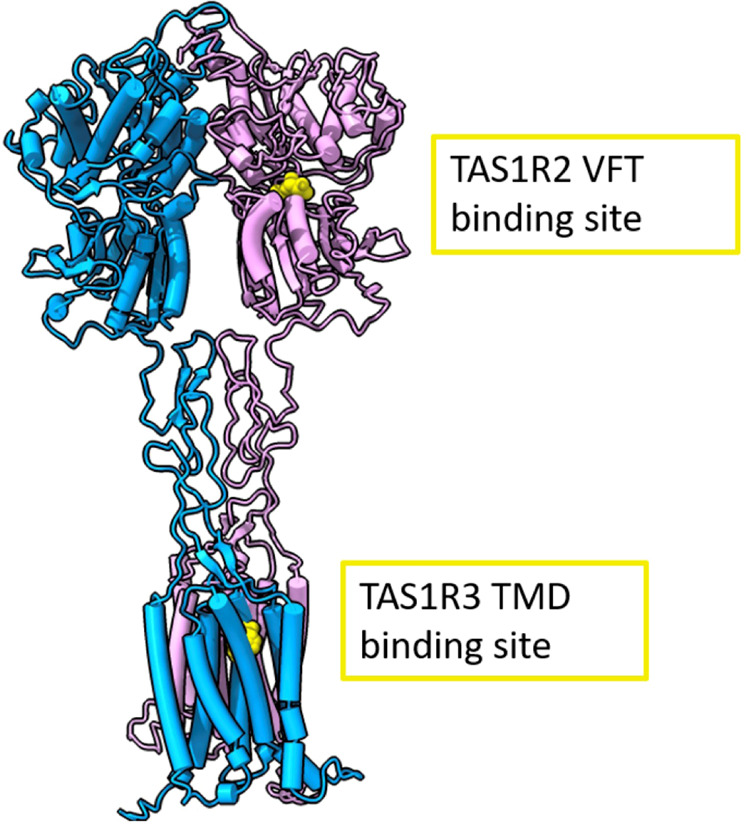
Full human sweet taste TAS1R2/TAS1R3 receptor model with the TAS1R2 monomer colored in pink and the TAS1R3 monomer in cyan. Binding sites are represented in yellow. The full receptor heterodimer was prepared with the I-Tasser web server^51^ based on multiple experimental structures (i.e., 6N51, 5X2M, and 5K5S). The binding site of TAS1R2 is based on coordinates of docked D-glucose to a Venus flytrap (VFT)^59^ (modeling based on template PDB ID: 5X2M, docking performed with Schrödinger Maestro 2019-1, Glide XP), and the TAS1R3-binding site is based on a lactisole molecule docked to the TAS1R3 TMD model (template PDB IDs: 4OR2 and 4OO9, Schrödinger Maestro 2018-2, Glide SP). The figure was made using ChimeraX (version 0.93)^60^.

Within the last two decades, the heterodimer of the taste receptor of the TAS1Rs type of G protein-coupled receptors (GPCRs), denoted as TAS1R2/TAS1R3, was established as the main receptor for sweet taste^22^. The human TAS1R2/TAS1R3 heterodimer recognizes diverse natural and synthetic sweeteners^23^. The binding sites of the different types of sweeteners include an orthosteric site (a sugar-binding site in the extracellular Venus flytrap domain of TAS1R2) and several allosteric sites, including sites in the extracellular regions of the TAS1R2 and TAS1R3 subunits and in the transmembrane domain of TAS1R3^24,25^ (Fig. 1). Additional pathways for sweet taste recognition have also been suggested, involving glucose transporters and ATP-gated K^+^ channel^26,27^.

Interestingly, not all artificial sweeteners are recognized by rodents^28^. Differences in human and rodent responses to tastants, as well as sweetness inhibitors such as lactisole, have been useful for delineating the molecular recognition of sweet compounds—using human-mouse chimeric receptors, it was shown that the transmembrane domain (TMD) of human TAS1R3 is required for the activating effects of cyclamate^29^ and for the inhibitory effect of lactisole^30^.

A combination of TAS1R3 with another member of the TAS1R family, TAS1R1, results in a dimer that mediates umami taste, elicited by molecules such as glutamate or, in case of the rodent umami receptor, other L-amino acids^31^. Bitter taste is mediated by the taste 2 receptor (TAS2R) gene family^32^, a branch of Family A GPCRs^24^. The human genome has 25 *TAS2R* subtypes and over a thousand of bitter compounds are currently known^33^, with numerous additional bitter tastants predicted^34^.

In this study, we systematically address the question of sweet taste of heavy water by a combination of sensory experiments in humans, behavioral experiments in mice, tests on sweet taste receptor-transfected cell lines, and computational modeling including molecular dynamics (MD) simulations. This combined approach consistently leads to the conclusion that the sweet taste of pure D_2_O is a real effect for human subjects due to activation of the TAS1R2/TAS1R3 sweet taste receptor. While present simulations show, in accord with previous experiments^35^, that proteins are systematically slightly more rigid and compact in D_2_O than in H_2_O, the specific molecular mechanism of the heavy water effect on the TAS1R2/TAS1R3 receptor remains to be established.

## Results and discussion

### Water purity

We have paid great attention to the purity of the water samples, further degassing and redistilling under vacuum the purest commercially available D_2_O and H_2_O. The lack of non-negligible amounts of organic impurities was subsequently confirmed by gas chromatography with mass spectrometry analysis and by experiments with water samples at different levels of purification, see Supplementary Information (SI), Figures S1 and S2. This is extremely important—note in this context that “the vibrational theory of olfaction”, which suggested distinct perception of deuterium isotopes of odorants due to difference in their vibrational spectra^36^, has been refuted with some of the observed effects turning out to be due to impurities^37,38^.

### Experiments with a human sensory panel

A human sensory panel was employed to study the D_2_O taste. Triangle tests based on two samples of H_2_O and one sample of D_2_O (or vice versa), with random success rate of one-third, were presented to the panelists in a randomized order. Panelists were asked to pick the odd sample out—to smell only, to taste only (with a nose clips), or to taste with open nose. Our results show that humans perceive D_2_O as being clearly distinguishable from H_2_O based on its taste: In open nose taste test 22 out of 28 participants identified the odd sample correctly (*p* = 0.001), and in taste only test 14 out of 26 identified the odd sample correctly (*p* = 0.03). However, in smell-only triangle test, only 9 out of 25 panelists chose the odd sample correctly (*p* > 0.05). Data are summarized in Figure S3 in SI.

**Fig. 2:**
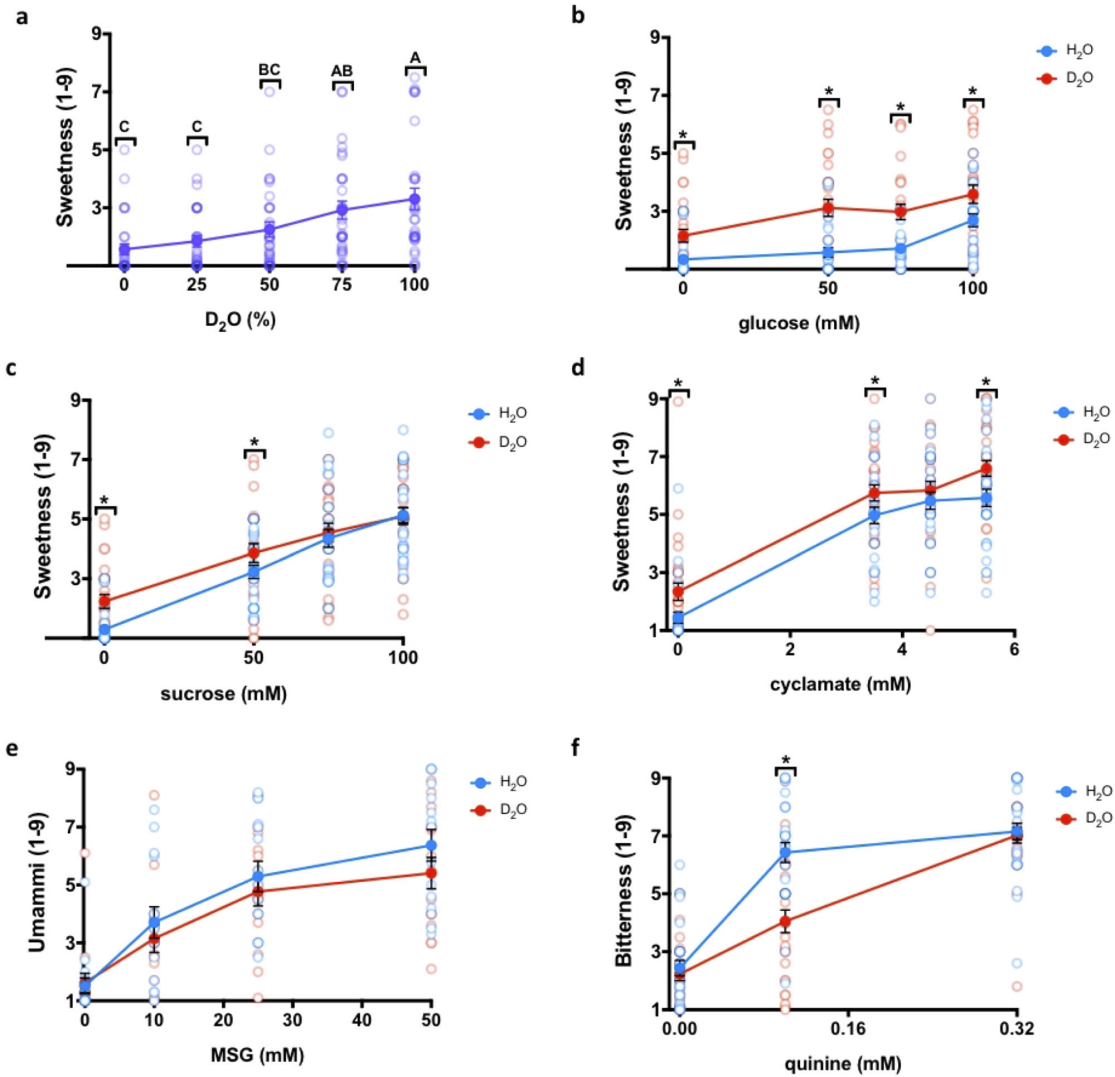
D_2_O sweetness and its effect on different tastants. **a** Sweetness of D_2_O mixed at increasing ratios with H_2_O. Treatments not connected by the same letters are significantly different (*p* < 0.05 in Tukey Kramer test). **b**–**f** The effect of D_2_O (red) compared to H_2_O (blue) on glucose (**b**), sucrose (**c**), cyclamate (**d**), quinine (**e**), and MSG (**f**) taste-specific intensity. Asterisks indicate a significant (*p* < 0.05) difference between water types using the two-way analysis of variance (ANOVA) with a preplanned comparison *t*-test. All data are presented as the mean ± the Standard Error of Measurement (SEM); *n* = 15–30 (4–12 males). The *y* axis shows the response for individual modalities, while the *x* axis is labeled with different water samples. Scale for each modality is labeled as 1 = no sensation, 3 = slight, 5 = moderate, 7 = very much, and 9 = extreme sensation.

Next, the perceived sweetness of D_2_O in increasing proportion to H_2_O was reported using a 9-point scale, labeled also with verbal descriptions of perceived intensity (1 = no sensation, 3 = slight, 5 = moderate, 7 = very much, 9 = extreme sensation). Sweetness was shown to increase in a D_2_O-dose-dependent manner, reaching average 3.3 ± 0.4 sweetness (“slight” sweetness) (Fig. 2a). The perceived sweetness of low concentrations of caloric D-glucose (Fig. 2b), sucrose (Fig. 2c), and an artificial sweetener cyclamate (Fig. 2d) was tested when dissolved in H_2_O or in D_2_O, in order to check whether the slight sweetness of D_2_O adds on top of slight sweetness of known sweeteners. As expected^39^, D-glucose was perceived as less sweet compared to sucrose at the same concentration (Fig. 2b and c). D_2_O added to the perceived sweetness of all tested concentrations of D-glucose and cyclamate (see Figure S4 in the SI for cyclamate results excluding two outliers). The sweetness of the two lowest concentrations of sucrose was significantly higher when dissolved in D_2_O compared to H_2_O.

We then checked whether the stand-alone and additive effect of D_2_O is sweetness-specific or general, whereby D_2_O might elicit other tastes, or add to their intensity. Savory (umami) taste of monosodium glutamate (MSG) and bitter taste of quinine, which are also taste modalities mediated by GPCR receptors expressed in taste cells, were tested in regular and in heavy water. The intensity of savory taste of MSG in D_2_O did not differ from that in H_2_O (Fig. 2e), while the perceived bitterness of quinine was in fact slightly reduced in D_2_O compared to quinine in H_2_O (Fig. 2f). This is in agreement with the known effect of sweeteners as maskers of bitter taste, that may be due to both local interactions and sensory integration effects^40–42^. Thus, we have ascertained that D_2_O is sweet and adds to the sweetness of other sweet molecules, but not to the intensity of other GPCR-mediated taste modalities.

### Experiments with mice

Next, we addressed the question whether the sweetness of D_2_O is perceived also by rodents. Lean mice of the C57BL/6J strain were drinking pure H_2_O, D_2_O, or a 43 mmol/l H_2_O sucrose solution for 16 h during a night period. Namely, each of the three groups of mice had a choice from two bottles containing (i) H_2_O and D_2_O, (ii) H_2_O and sucrose solution, or (iii) H_2_O and H_2_O (as a control). The food intake was unaffected in all groups (see SI, Figure S5 and Table S1).

**Fig. 3:**
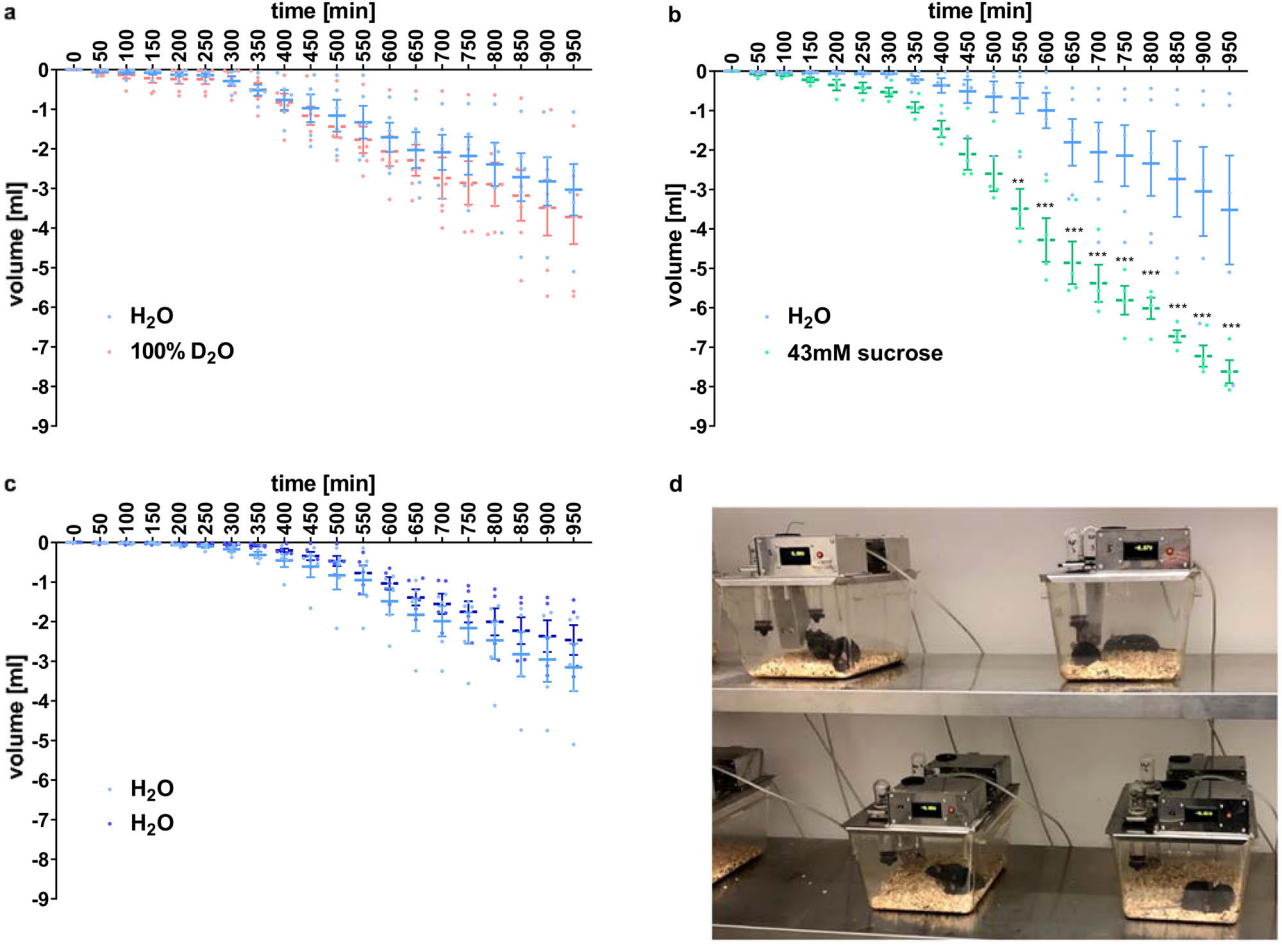
Time-resolved volumes of water consumption by mice. **a** Volume consumption of D_2_O is not different from that of H_2_O (*n* = 12). **b** Mice show strong preference to sucrose solution (*n* = 10). Significance is **p < 0.01, ***p < 0.001. **c** Volume consumption of the control group drinking H_2_O only (*n* = 12). **d** Snapshot of the automatic drinking monitoring system. Mice were placed in groups of two in individual cages. Data are presented as the mean ± standard error of the mean (SEM). Statistical analysis was performed using two-way ANOVA with a Bonferroni’s multiple comparisons test.

The results of the drinking experiments are presented in Fig. 3a–c, with a snapshot of the experimental setup shown in Fig. 3d. In cages where mice were offered both normal water and heavy water (Fig. 3a) consumption of D_2_O was within statistical error the same as that of H_2_O. Previous reports have shown that on longer timescales than those reported here mice learned to avoid D_2_O, as it is poisonous to them in larger quantities^10^. It is not clear what is the cue that enables the avoidance learning, but it is evident that the early response to D_2_O is not attractive, suggesting that it is not eliciting sweet taste in mice.

By contrast, mice exhibit a strong preference for sucrose solution over H_2_O. Indeed, the consumed volume was significantly increased in line with the predilection of mice for sucrose solutions (Fig. 3b). The amount of H_2_O consumed by the control group from either of the two bottles, both containing H_2_O, is depicted in Fig. 3c. Overall, the data shows that in all three experiments mice consumed comparable amounts of H_2_O and D_2_O, with significant increase of consumption of the sucrose solution.

### Assessing involvement of TAS1R2/TAS1R3 receptor using human sensory panel

**Fig. 4:**
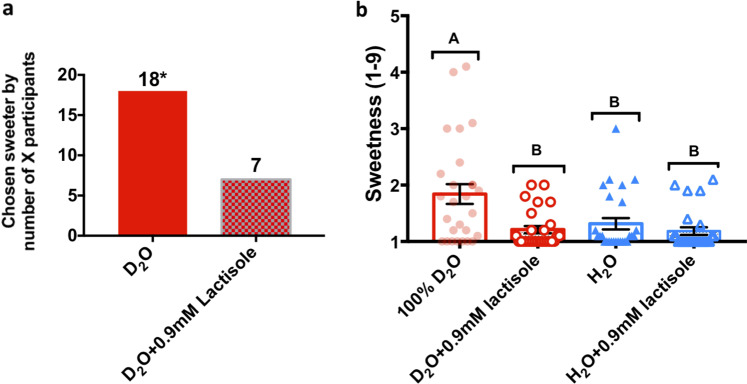
Lactisole reduces sweetness of D_2_O. **a** 2AFC test. Pure D_2_O was chosen to be sweeter (*p* < 0.05) than the sample with lactisole by 18 participants (*n* = 25; 11 males). **b** Effect of 0.9 mM lactisole on sweetness intensity using the 9-point scale. Data are presented as the mean ± SEM. The *y* axis shows the response for sweetness on a 9-point scale, while the *x* axis is labeled with different water samples. Statistical analysis was performed using ANOVA with a Tukey Kramer test (*n* = 27; 9 males); treatments not connected by the same letters are significantly different (*p* < 0.05). Scale for sweetness is labeled as 1 = no sensation, 3 = slight, 5 = moderate, 7 = very much, and 9 = extreme sensation.

The chemical dissimilarity of D_2_O from sugars and other sweeteners raises the question whether the effect we observed in human subjects is mediated by TAS1R2/TAS1R3, which is the major receptor for sweet taste^22^. This was first explored by combining water samples with lactisole as an established TAS1R2/TAS1R3 inhibitor^30^. Using the two-alternative forced-choice (2AFC) method, in which the participant must choose between two samples, 18 out of 25 panelists chose pure D_2_O as sweeter than D_2_O + 0.9 mM lactisole solution (*p* < 0.05, Fig. 4a). In an additional experiment, the sweetness of pure D_2_O was scored significantly higher than that of D_2_O + 0.9 mM lactisole solution (*p* = 0.0003), while the same amount of lactisole had no effect on the perception of sweetness of H_2_O that served as control (Fig. 4b). These results suggest that D_2_O elicits sweetness via the TAS1R2/TAS1R3 sweet taste receptor.

### Cell-based experiments for establishing the role of TAS1R2/TAS1R3

**Fig. 5:**
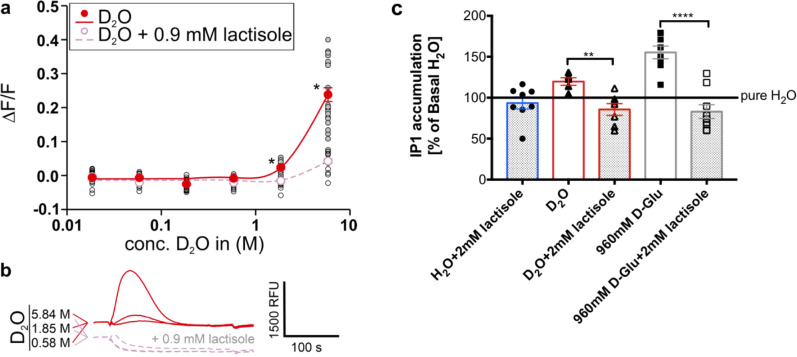
D_2_O-activation of the human sweet taste receptor. **a** Dose–response relationship of cells expressing the human sweet taste receptor and treated with different concentrations of D_2_O (filled red circles, red line). Cells treated with lactisole served as negative controls (open pink circles, pink line). *y* axis, relative changes in fluorescence upon stimulus application (Δ*F*/*F*). *x* axis, logarithmically scaled molar D_2_O-concentrations. Asterisks indicate fluorescence changes above baseline significantly different from lactisole-treated controls (*p* ≤ 0.01). **b** Raw fluorescence traces of D_2_O-treated (red-traces, top) and D_2_O + 0.9 mM lactisole-treated cells (pink-traces, bottom) stimulated with the indicated D_2_O-concentrations. A scale bar indicating relative fluorescence (relative fluorescence units (RFU) and experimental time (in seconds (s)) is included. **c** Lactisole inhibitory effect on D_2_O-activation of the human sweet taste receptor using an IP1 assay. Cells treated with D-glucose served as positive controls. On *y* axis, relative changes in IP1 accumulation upon stimulus application are shown as % of basal–pure H_2_O. *x* axis, different ligands, with and without lactisole. Asterisks indicate IP1 changes that are significantly different from lactisole-treated controls (**p ≤ 0.005 and ****p ≤ 0.0001) using *t*-test.

To confirm the involvement of the sweet taste receptor TAS1R2/TAS1R3 in D_2_O signaling we performed functional calcium mobilization assays using HEK 293 FlpIn T-Rex cells heterologously expressing both required TAS1R subunits as well as the chimeric G protein Gα15Gi3^43,44^. As seen in Fig. 5a, D_2_O at 1.85 M and 5.84 M concentrations in H_2_O (3.3% and 10.4%, respectively) elicited robust responses in TAS1R2/TAS1R3 expressing cells. The strong reduction or absence of D_2_O-elicited fluorescence response in the presence of lactisole confirmed the dependence on TAS1R2/TAS1R3. The inhibitory effect of lactisole on D_2_O-activation of the human sweet taste receptor was confirmed using an IP1 assay^45,46^, while lactisole exposure had no effect on cells treated with pure H_2_O water, as expected (Fig. 5c). As a control, 960 mM D-glucose elicited increase in IP1 levels in TAS1R2/TAS1R3 expressing cell, which was inhibited in the presence of lactisole.

**Fig. 6:**
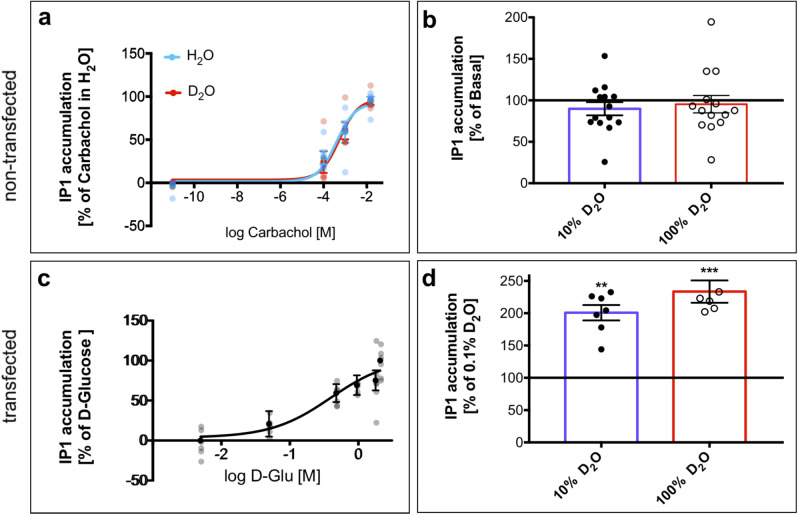
IP1 accumulation in HEK 293T cells following exposure to different ligands dissolved in powder-based DMEM medium. **a** Non-transfected HEK 293T cells respond similarly to raising concentrations of carbachol dissolved in D_2_O (red) as in H_2_O (blue). **b** D_2_O caused no elevation of IP1 levels in non-transfected HEK 293T cells. **c** HEK 293T cells transiently expressing TAS1R2/TAS1R3 respond positively to D-glucose. **d** Transfected HEK 293T cells are activated by D_2_O. Values represent the mean ± SEM of at least three replicates. The horizontal black line represents the basal values of controls. Significant differences in IP1 values from control values are marked with **p ≤ 0.005 and ***p ≤ 0.0005 using Dunnett’s multiple comparisons test.

We further used an IP1 assay^45,46^ on non-transfected HEK 293T cells, where we observed that dose-dependent curves of carbachol—an agonist of the endogenous muscarinic receptor 3 (M3)^47^—did not show any difference between H_2_O and D_2_O-based media (Fig. 6a) and that cell medium that had either 10% or 100% D_2_O, did not activate basal IP1 accumulation (Fig. 6b). Next, TAS1R2/TAS1R3 receptor along with the chimeric Gα16gust44 subunit^44,48^ were transiently expressed, and the functionality was illustrated by dose-dependent response to D-glucose (Fig. 6c). Finally, and in agreement with calcium imaging, we found that 10% D_2_O activated these cells. Activation by 100% D_2_O was even more pronounced (Fig. 6d).

### Molecular modeling

**Fig. 7:**
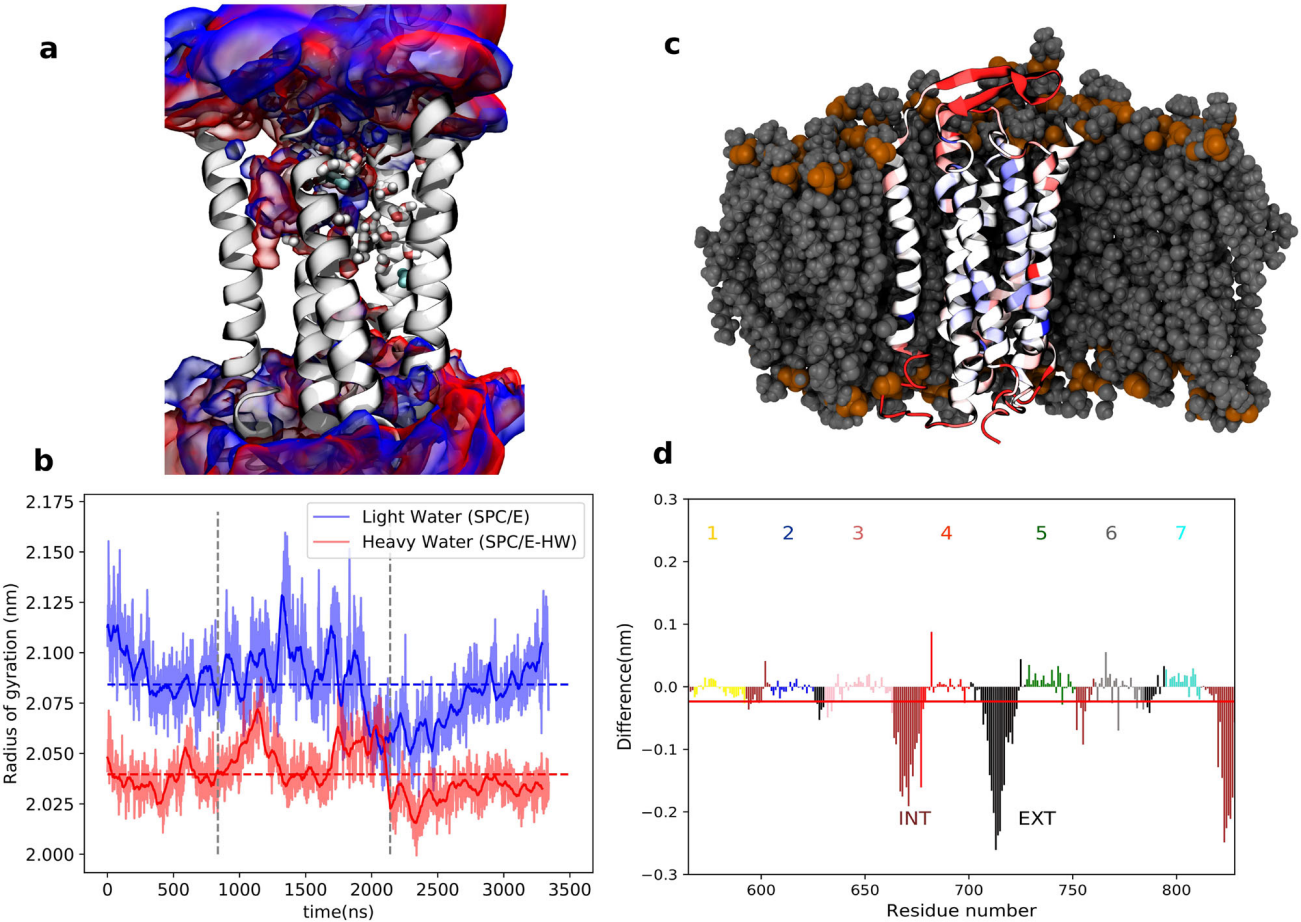
Differences between the behavior of the transmembrane part of the human sweet taste receptor in H_2_O vs D_2_O base on analysis of three independent microsecond trajectories. **a** Structure of the TMD of the TAS1R2/TAS1R3 receptor with the probability density (volumetric map) of H_2_O (blue) or D_2_O (red) molecules within 10 Å from the protein evaluated using the VMD VolMap tool from the MD simulations at an isovalue of 0.1. The conserved water molecules in the X-ray templates are shown in cyan color. Water molecules predicted with the software OpenEye^52^ are shown in licorice representation. **b** Time evolution of the radii of gyration in H_2_O (blue) and D_2_O (red) from three microsecond-timescale simulations (separated by vertical dashed lines) with total mean values as dashed lines, showing that the protein is more compact in heavy water. **c** Representative snapshot of the transmembrane part of the human sweet taste receptor color-coded that red/blue represents parts more/less rigid in D_2_O vs H_2_O. The embedding lipid membrane is represented in gray. **d** Difference in root mean square fluctuations in MD trajectories. Negative/positive values mean that structures are more/less rigid in D_2_O than in H_2_O. The red line represents the sum over all residues. INT intracellular, EXT extracellular.

The cellular response results further support the hypothesis that the sweet taste of D_2_O is mediated via the TAS1R2/TAS1R3 receptor. Various mechanisms governing this effect can be envisioned. As a potential suspect, we focus on a direct effect on the sweet taste receptor, narrowing on the TAS1R3 TMD (see Fig. 1), as it is already known to be a modulation site with functional differences between humans and rodents^23,29,30^. Furthermore, water-binding sites were discovered at the TMD of many GPCRs^49,50^, suggesting a potential target for D_2_O binding. We modeled the human TAS1R3 TMD using the I-TASSER server^51^. Positions of H_2_O molecules were compared among mGluR5 structures (PDB: 4OO9, 5CGC, and 5CGD) and two conserved positions were found. The H_2_O molecules in these two positions were merged with the TAS1R3 model and minimized (Fig. 7a). The water mapping protocol from OpenEye^52^ enables mapping of water positions based on the energetics of water, and ~40 water molecules were predicted in the binding site using this protocol (Fig. 7a). Water densities of H_2_O and D_2_O in the TMD of the TAS1R2/TAS1R3 receptor were calculated from MD simulations as described below. Overall, all three methods suggest the possibility for at least some internal molecules (trapped in the TMD bundle) in addition to water that surrounds the extracellular and intracellular loops (Fig. 7a).

Next, we carried out microsecond MD simulations of the TMD embedded in a phospatidylcholine (POPC) bilayer in either H_2_O or D_2_O (for details including our model of D_2_O effectively including nuclear quantum effects, see SI, Tables S2–S4). Note that water molecules enter the TMD domain and cluster at positions that partially overlap with the modeled water positions, see Fig. 7a. More precisely, H_2_O and D_2_O have mutually slightly shifted densities inside the protein cavity, with H_2_O overlapping better than D_2_O with the modeled water positions. Furthermore, MD simulations show clustered water molecules close to the lactisole binding site. These internal positions may have a differential effect between H_2_O and D_2_O, though differences between the averaged water densities are not very pronounced. Figure 7b shows the time evolution of the radius of gyration of the TMD domain, while Fig. 7c and d presents the root mean square fluctuations (RMSF) of individual residues of the proteins superimposed on its structure and plotted in a graph together with the mean value of RMSF. A small but significant difference is apparent in the behavior of the protein in H_2_O vs D_2_O. Namely, structural fluctuations of most residues (particularly those directly exposed to the aqueous environment) and of the protein as a whole are slightly attenuated in D_2_O, in which environment the protein is also somewhat more compact than in H_2_O (Fig. 7b). Additional simulations on other representative systems show that the rigidifying effect of heavy water is apparent also in small soluble proteins (see SI, Figs. S6–S8).

### Summary and outlook

Sweet taste never ceases to surprise. Over a decade ago, water was shown to elicit sweet taste by rinsing away inhibitors of sweet taste receptors, both in human sensory experiments and in cell-based studies. This effect was explained in terms of a two-state model, where the receptor shifts to its activated state when released from inhibition by rinsing with water^43^. Here, we have studied the taste of D_2_O and H_2_O per se, not related to washing away of sweet taste inhibitors. Using psychophysics protocols, we show that humans differentiate between D_2_O and H_2_O based on taste alone. Importantly, by employing gas chromatography/mass spectrometry analysis we demonstrate that the sweet taste of deuterated water is not due to impurities. Being only isotopically different from H_2_O, in principle, D_2_O should be indistinguishable from H_2_O with regard to taste, namely it should have no taste of its own. Yet, we illustrate that human subjects consistently perceive D_2_O as being slightly sweet and significantly sweeter than H_2_O. Furthermore, D_2_O added to perceived sweetness of low concentrations of other sweeteners. In contrast, it did not elicit umami or bitter taste on its own, nor did it add to the umami taste perception of MSG. D_2_O did not add to the bitterness of quinine, and reduced the perceived bitterness of 0.1 mM quinine, in agreement with the known effect of bitterness suppression by sweet molecules.

A further important finding is that lactisole, which is an established blocker of the TAS1R2/TAS1R3 sweet taste receptor that acts at the TAS1R3 transmembrane domain^30^, suppresses both the sweet perception of D_2_O in sensory tests, as well as the activation of TAS1R/TAS1R3 in calcium imaging and in IP1 cell-based assays. In support of these observations we have demonstrated that HEK 293T cells transfected with TAS1R2/TAS1R3 and Gα16gust44 chimera, but not the non-transfected cells, are activated by D_2_O, as measured by IP1 accumulation compared to control values. Finally, taste experiments on mice show that these animals do not prefer D_2_O over H_2_O.

Our findings point to the human sweet taste receptor TAS1R2/TAS1R3 as being essential for sweetness of D_2_O. Molecular dynamics simulations show, in agreement with experiment^35^, that proteins in general are slightly more rigid and compact in D_2_O than in H_2_O. At a molecular level, this general behavior may be traced back to the slightly stronger hydrogen bonding in D_2_O vs H_2_O, which is due to a nuclear quantum effect, namely difference in zero-point energy^3,4^. Biologically relevant situations where one may expect strong nuclear quantum effects as implications of H/D substitution directly involve proton or deuteron transfer^9^. Unless a yet unknown indirect mechanism is involved, this is not the case for the TAS1R2/TAS1R3 sweet taste receptor, thus the nuclear quantum effect is probably weak in the present case. Future studies should be able to elucidate the precise sites and mechanisms of action, as well as the reason why D_2_O activates TAS1R2/TAS1R3 in particular, resulting in sweet (but not other) taste. To this end, site-directed mutagenesis as well as determination of the precise structure of the TAS1R2/TAS1R3 receptor will be of key importance.

The finding that deuterated water elicits sweet taste via activation of TAS1R/TAS1R2 receptor is of fundamental interest. The difference between hydrogen isotopes is the largest possible isotope effect (doubling of mass in case of deuterium, while tripling in case of tritium), yet deuteration effects on water are generally mild. Nevertheless, water deuteration leads to activation of a GPCR heterodimer to a level that is perceived by humans as sweet taste. While clearly not a practical sweetener, heavy water provides a glimpse into the wide-open chemical space of sweet molecules. Since heavy water may be used in medical procedures, our finding that it can elicit responses of the sweet taste receptor, which is not only located on the tongue but also in other tissues of the human body, represents an important information for clinicians and their patients. Moreover, due to wide application of D_2_O in chemical structure determination by NMR, chemists will benefit from being aware of the present observations.

## Methods

### Sensory evaluation experiments

A human sensory panel was used to resolve the gustatory effect in perception of D_2_O taste. Subjects between the ages 20 and 43 years were recruited. The study included 10 experiments with different groups of participants (15–30 subjects; between 4 and 12 males). The perception was tested by sensory tests as detailed below. Either sterile syringes with solutions (0.3 ml) or identical cups with solutions (7 ml), were presented in randomized order, unless otherwise noted. Participants were required to taste each solution using either ‘tip of the tongue’ or ‘sip and spit’ procedures, rinse their mouth with water after each solution and to wait for 30 s before moving to the next taste sample. All research procedures were ethically approved by the Committee for the Use of Human Subjects in Research in The Robert H. Smith Faculty of Agriculture Food and Environment, the Hebrew University of Jerusalem.

99.9% purity D_2_O was purchased from Sigma-Aldrich Corp, while 18 MΩ ultrapure grade was used for pure H_2_O. All water samples were placed under vacuum and treated by ultrasound to remove any dissolved gases. See below for more details on water purification.

D-glucose (CAS Number: 50-99-7), sucrose (CAS Number: 57-50-1), cyclamate (CAS Number: 139-05-9), quinine (CAS Number: 207671-44-1), and MSG (CAS Number: 142-47-2) were purchased from Sigma-Aldrich Corp. All compounds were dissolved in both types of water to a final concentration of 50, 75, and 100 mM for D-glucose and sucrose; 3.5, 4.5, and 5.5 mM for cyclamate; 0.1 and 0.32 mM for quinine; 10, 25, and 50 mM for MSG. Lactisole (CAS Number: 150436-68-3) was purchased from Domino Specialty Ingredients and dissolved to a final concentration of 0.9 mM in D_2_O water as well as in H_2_O. The concentration of lactisole was selected based on previous data^43,53^ and preliminary experiments in our lab, which showed that 0.9 mM decreases the sweetness of 100 mM sucrose. Sweeteners concentrations were selected to be in low intensity of sweetness. All solutions were prepared in the morning of the day of the experiment and were stored in individual plastic syringes (1 ml) for each participant.

The sweetness of heavy water and its effect on other taste compounds was evaluated in several independent experiments: (1) D_2_O sweetness relative to H_2_O (Fig. 2a); (2) D_2_O effect on sweetness of D-glucose (Fig. 2b), sucrose (Fig. 2c), and cyclamate (Fig. 2d); (3) D_2_O effect on quinine (Fig. 2e) and MSG (Fig. 2f); (4) Lactisole effect on D_2_O sweetness (Fig. 4b); Intensity of each taste modality—sweetness/bitterness/umami was evaluated on a 9-point scale on Compusense Cloud, ranging from 1 (no sensation) to 9 (extremely strong sensation). In addition, participants had to report any additional tastes they recognized. Statistical tests were conducted using JMP Pro 13 (JMP, Version 13. SAS Institute Inc., Cary, NC, 1989–2019).

Data were first analyzed employing ANOVA with participants as a random effect^54^. Thereafter, the Tukey Kramer test was used to compare mean sweetness between all samples^54^. Significance was set at *p* < 0.05, and preplanned comparison *t*-tests were used where relevant.

Details concerning the Two-Alternative Forced Choice test^55^ were as follows: Participants were presented with two blind coded water samples of D_2_O and D_2_O + 0.9 mM lactisole. The participants were asked to choose the sweeter solution. For the data analysis, the highest number of responses for one sample was compared to a statistical table^55^ which states the minimum number of responses required for a significant difference.

Panelists were presented with two identical and one different water samples. All three samples were presented to the subjects at once, and the panelists were instructed to taste or smell the samples from left to right and identify the odd sample. Triangle tests were used to examine the difference in taste, as well as in smell, between H_2_O and D_2_O.

### Heterologous expression

TAS1R2/TAS1R3 stimulated activation of the G-protein-mediated pathway was measured applying the IP-One HTRF assay (Cisbio) based on the manufacturer’s protocol. In brief, HEK 293T cells (ATCC) were grown to a confluency of ~85–90% and transiently transfected with 6 μg/plate DNA (TAS1R2, TAS1R3, Gα16gust44) by applying LipofectamineTM 2000 (Invitrogen, USA, 30 μl/plate) transfection reagent, according to the manufacturer’s protocol. The next day, cells were suspended with fresh Dulbecco’s modified Eagle’s medium (DMEM), containing 10% fetal bovine serum (FBS), 1% L-glutamine amino acid and 1% penicillin streptomycin (10% DMEM), seeded (0.5 ml cells per well) into 24-well culture plate, and maintained for 8–12 h at 37 °C. Then cells were “starved” overnight by changing the medium to 0.1% DMEM (containing 0.1% FBS), in order to reduce the basal activity of the cells. Cells exposure was performed by addition of 0.5 ml tested compound (pH = 7.4) dissolved in 0.1% DMEM with 50 mM lithium chloride (LiCl) for 5 min directly into the wells. The presence of LiCl in this step is crucial because LiCl leads to IP1 accumulation^45^. At the end of exposure time, tastant solution was replaced with fresh medium (0.1% DMEM) containing 50 mM LiCl for another 55 min. Later, wells were washed with 100 μl cold phosphate-buffered saline (PBS) + Triton X-100, and kept at −80 °C for a few hours, in order to dissolve the cell membrane. For the IP-One HTRF assay, cell lysate was mixed with the detection reagents (IP1-d2 conjugate and Anti-IP1 cryptate TB conjugate, each dissolved in lysis buffer), and added to each well in a 384-well plate for 60 min incubation at room temperature. Finally, the plate was read using Clariostar plate reader (BMG, Germany) equipped with 620 ± 10 nm and 670 ± 10 nm filters. IP1 levels were measured by calculating the 665 nm/620 nm emission ratio.

All responses are presented as the means ± SEM of IP1 accumulation (%). Dose–response curves were fitted by non-linear regression using the algorithms of PRISM 7 (GraphPad Software, San Diego, CA, USA). Column figures were analyzed using one-way ANOVA with a Dunnett’s^54^. Each compound was tested in triplicate in three individual experiments in comparison to the reference (carbachol dissolved in H_2_O or basal levels)^45^.

In the case of D_2_O, in order to test its specific effect, we used a powder DMEM medium (CAS Number: D5030, Sigma-Aldrich), dissolved in the needed amount of D_2_O instead of the liquid one. Other ligands than D_2_O were purchased from Sigma-Aldrich or Domino Specialty Ingredients as noted above. Unless noted otherwise, ligands were used at final concentrations of 5, 50, 480, 960, 1.8 × 10^3^, and 2.1 × 10^3^ mM for D-glucose; 0.1, 1, and 15 mM for carbachol and D_2_O at 0–100% proportionately to H_2_O. Since there was no prior literature on concentrations of lactisole for this assay, the value of 2 mM was chosen based on preliminary experiment with several lactisole concentrations and their effect on IP1 values due to exposure to D-glucose.

For the functional assays with the human sweet taste receptor, we used a cell line (HEK 293 FlpIn T-Rex), which constitutively expresses the sweet taste receptor subunit TAS1R2 as well as the chimeric G protein Gα15gi3, whereas the sweet taste receptor subunit TAS1R3 can be induced by tetracycline^43,44^. Gαi3 is one of the major species of Gα-subunits capable of coupling to taste receptors, along with Gα-gustducin, GαS, and Gαi2^56,57^ and TAS1R2-TAS1R3-Gαi3 cell system was successfully used in a previous study^43^. The functional experiments were done as described before^44^. Briefly, the cells were grown in DMEM supplemented with 10% fetal bovine serum, 100 U Penicillin/ml, 0.1 mg/ml Streptomycin, 2 mM L-glutamine, at 37 °C and 5%-CO_2_, 100% air humidity. The day before the experiment, cells were seeded to a density of 50–60% onto 96-well plates coated with 10 µg/ml poly-D-lysine and 0.5 µg/ml tetracycline was added. Next, cells were loaded with Fluo-4 AM in the presence of 2.5 mM probenecid for 1 h. After this, cells were washed twice with C1-buffer (130 mM NaCl, 5 mM KCl, 10 mM HEPES, 1 mM sodium pyruvate, and 2 mM CaCl_2_, pH 7.4) before placing them in a fluorometric imaging plate reader (FLIPR^tetra^, Molecular Devices) for measurements.

C1-buffer prepared with D_2_O was mixed with C1-buffer made with H_2_O to result in the following final D_2_O-concentrations (a further threefold dilution, which occurs upon application of 50 µL stimulus to 100 µL of C1-buffer in the 96-well plates is already included): 18.47, 5.84, 1.85, 0.584, 0.185, 0.058, 0.018, and 0.000 M. Fluorescence changes were monitored after automated application of stimuli. As specificity control C1-D_2_O including 0.9 mM lactisole, a selective inhibitor of the human sweet taste receptor^58^, was applied to identically treated cells. This concentration is the same as used in sensory experiments and close to the 1 mM used in calcium assays in previous work^43^. Experimental results from five biological replicates performed in quadruplicates were used to establish the dose–response relationship using the software SigmaPlot as before^44^. As the highest D_2_O-concentration resulted in fluorescence changes largely resistant to lactisole blocking, the 18.47 M concentration was excluded. Student’s *t*-test was used to confirm that D_2_O-induced fluorescence changes above baseline were significantly (*p* ≤ 0.01) different from lactisole-treated controls.

### Animal experiments

All animal experiments followed the ethical guidelines for animal experiments and the Act of the Czech Republic Nr. 246/1992 and were approved by the Committee for Experiments with Laboratory Animals of the Czech Academy of Sciences. Three-month-old male C57BL/6J mice (*n* = 34) from Charles Rivers Laboratories (Sulzfeld, Germany) were housed at a temperature of 23 °C with a daily cycle of 12 h light and dark (lights on at 6 am). The mice were placed in groups of two in cages with automatic drinking monitoring system (Developmental Workshops of Institute of Organic Chemistry and Biochemistry of the Czech Academy of Sciences, Prague, Czech Republic). They were given ad libitum water and a standard rodent chow diet (Ssniff Spezialdiäten GmbH, Soest, Germany). On the day of the experiment, during the dark phase of the cycle, freely fed mice were given weighed food pellets and two 30 ml glass bottles. The two bottles contained pure H_2_O and pure D_2_O (*n* = 12), or H_2_O and sucrose solution (43 mmol/l sucrose solution in H_2_O) (*n* = 10), see Table S1. Mice drinking H_2_O in both bottles served as a control group (*n* = 12). Drinking was monitored every 10 min for 16 h (starting from 6 pm) and food intake was determined at the end of the experiment (Figure S5).

All responses are presented as the means ± SEM. Statistical analysis was performed using ANOVA with a Dunnett’s test^54^ for food intake. Two-way ANOVA with a Bonferroni’s multiple comparisons test was used for analysis of average volume of liquid consumption. Analysis was performed using GraphPad Software, Inc., Prism 8 (GraphPad Software, San Diego, CA, USA). The differences between the control and treated groups were considered significant at *p* < 0.05.

### Modeling and docking

All models were prepared with I-Tasser web server^51^. The templates that were used by I-Tasser for each of the TAS1R2 and TAS1R3 monomers were: 6N51, 5X2M, and 5K5S. To model the full heterodimer, the monomer models were aligned to a Class-C GPCR Cryo-EM structure (PDB: 6N51) and minimized with Schrödinger Maestro 2019-1. To illustrate the orthosteric binding site of sugars D-glucose was prepared (Schrödinger Maestro 2019-1, LigPrep) and docked with Glide XP, to the TAS1R2 VFT domain model that was based on 5X2M in a protocol that was validated in previous work^59^. The TAS1R3-binding site is based on a lactisole molecule docked to TAS1R3 TMD model (Schrödinger Maestro 2018-2, Glide SP, template PDB ID: 4OR2 and 4OO9). The figure was made using ChimeraX (version 0.93)^60^. Water molecules were predicted with a water mapping software (SZMAP 1.5.0.2: OpenEye^52^) after a successful benchmark over conserved water templates (PDB IDs 5CGC and 5CGD), in which the software was able to identify overall 8 out of 10 crystal water molecules around the ligands of the templates.

### Reporting summary

Further information on research design is available in the Nature Research Reporting Summary linked to this article.

## Supplementary information

Supplementary Information

Description of Supplementary Files

Supplementary Data 1

Supplementary Data 2

Supplementary Data 3

Supplementary Data 4

Supplementary Data 5

Supplementary Data 6

Supplementary Data 7

Supplementary Data 8

Reporting Summary

## Data Availability

The data sets generated during the current study are available as Supplementary Data.
