# Supplementary material for: Sweet taste of heavy water
Source: Commun Biol. 2021 Apr 6;4:440. doi: 10.1038/s42003-021-01964-y (PMC8024362; doi:10.1038/s42003-021-01964-y)
Supplement: Supplementary file 1 — Supplementary Information [file 42003_2021_1964_MOESM1_ESM.pdf]

Supplementary Information:

Sweet taste of heavy water

Natalie Ben Abu[1+], Philip E. Mason[2+], Hadar Klein[1], Nitzan Dubovski[1], Yaron Ben Shoshan-Galeczki[1], Einav Malach[1], Veronika Pražienková[2], Lenka Maletínská[2], Carmelo Tempra[2], Victor Cruces Chamorro[2], Josef Cvačka[2], Maik Behrens[3], Masha Y. Niv[1*] and Pavel Jungwirth[2*]

[1]The Institute of Biochemistry, Food Science and Nutrition, The Robert H Smith Faculty of Agriculture, Food and Environment, 76100 Rehovot, Israel and The Fritz Haber Center for Molecular Dynamics, The Hebrew University of Jerusalem, 91904 Jerusalem, Israel, [2]Institute of Organic Chemistry and Biochemistry, Czech Academy of Sciences, Flemingovo nám. 2, 16610 Prague 6, Czech Republic, and [3]Leibniz-Institute for Food Systems Biology at the Technical University of Munich, Lise-Meitner-Str. 34, 85354 Freising, Germany.

[+]Both authors contributed equally.

[*]Corresponding authors: masha.niv@mail.huji.ac.il and pavel.jungwirth@uochb.cas.cz

Details of the water purification procedure

Water purification method was designed to achieve two things: i) to remove all volatile components from water and ii) remove all non-volatile components. An all borosilicate glass apparatus with a 250 ml flask and a side arm with five takeoff points for ampoules was created. The apparatus was completely washed three times with 18 MΩ Millipore water and dried under vacuum. The glassware was flamed under vacuum to 400 C. This has the effect of burning off any volatiles in the apparatus. The kit was then charged with 180 ml of H_2O (18 MΩ Millipore) or D_2O (Sigma-Aldrich, 99.9 %). Water was then pumped out under vacuum to a teflon headed pump for 10 minutes while simultaneously being sonicated, which removes any dissolved gas. The flask was then heated to 60 °C. Water was then 'distilled' (vapor transported) to the fifth ampoule submerged in iced water. After 5 ml of water were condensed, this ampoule was sealed

with a glass torch and discarded. The remaining water was distilled into four 40 ml ampoules (using again iced water), each being sealed under vacuum with a glass torch. The remaining water in the apparatus was discarded. The same apparatus and procedure were used for both H_2O and D_2O.

The purity of H_2O and D_2O was further checked by gas chromatography coupled to mass spectrometry (GC/MS). The organic impurities that may have been present in the water were extracted with hexane (distilled in a glass apparatus from analytical grade solvent supplied by Penta, Czech Republic). A sample of water (6 ml) was shaken vigorously for 5 minutes with 2 ml of hexane in a ground-glass stoppered test tube. The organic layer was collected and its volume was reduced to 200 μl under a stream of nitrogen. The analyses were performed on a 6890N gas chromatograph coupled to a 5975B quadrupole mass spectrometer (Agilent Technologies, Santa Clara, CA). The sample (1 μl) was injected in the split mode with a split ratio of 10:1 and with the injector temperature set to 200 °C. An HP-5MS fused silica capillary column (30 m × 250 μm; a film thickness of 0.25 μm) from Agilent Technologies was used for the chromatographic separation. The carrier gas was helium at a constant flow rate of 1.0 ml/min and the temperature program was set as follows: 40 °C (2 min), then 8°C/min to 200°C (0 min), then 15°C/min to 320°C (3 min). The transfer line, ion source, and quadrupole temperatures were set to 280 °C, 230°C, and 150°C, respectively. The ionization was achieved using 70 eV electrons and the solvent delay time of 4 min was used. The total ion current chromatograms (*m/z* 29 - 600 range) shown in Figure S1 displayed background, without any signs of chromatographic peaks. Therefore, it can be concluded that the water samples used for the experiments did not contain significant amounts of volatile or semi-volatile organic compounds.

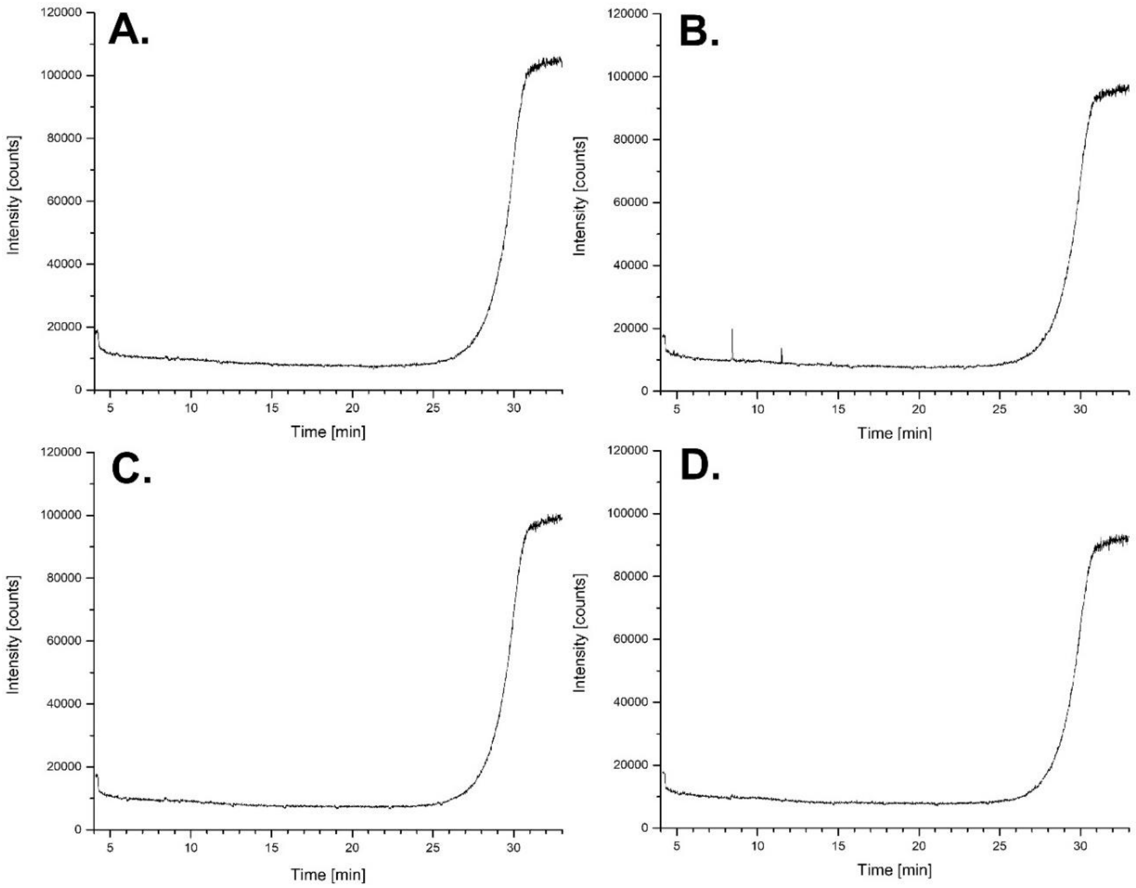

Figure S1. GC/MS chromatograms of hexane extracts of H_2O (A., B.) and D_2O (C., D.) showing clean background, without any chromatographic peaks. The background signals have typical profiles, with a tail of the solvent peak (4 min) and a rising signal at high retention times due to stationary phase bleeding at elevated temperatures. Small signals in panel B. are not chromatographic peaks but spikes caused by air occasionally penetrating the detector through the instrument fittings.

Ruling out impurity effects on taste

As seen in Figure S2, D_2O was rated sweeter than H_2O for all D_2O and H_2O purities that were tested, i.e., from the first, second, third, and fourth distillation batch. Here we used the labeled magnitude scale (LMS) for sweetness evaluation. Each participant (n=28; 8 males) was asked to taste eight solutions, four per each type of water, which differed in their level of purity. The solutions were offered in order of ascending purity

and randomized in terms of D_2O and H_2O. The scale was bound by ‘no sensation’ at the bottom and ‘strongest imaginable’ at the top of the range(*1*).

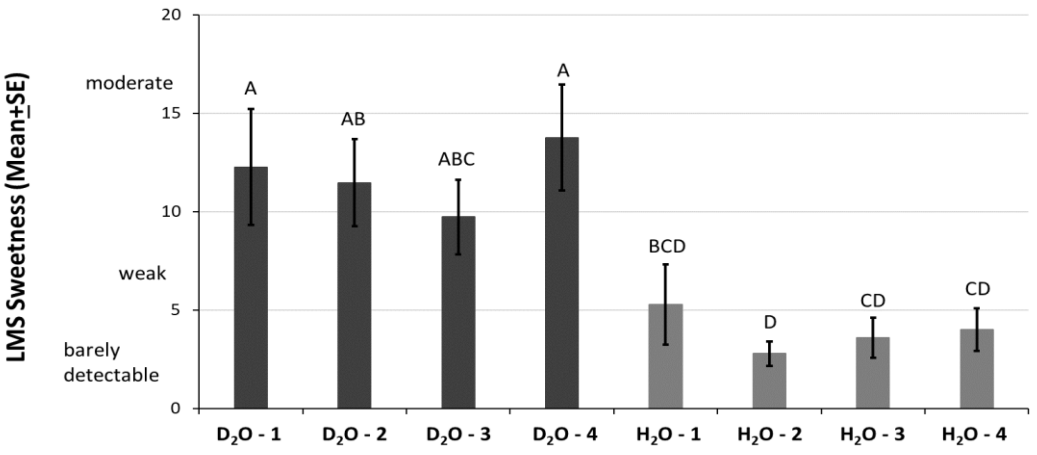

Figure S2. Difference in sweetness between two types of water at the same level of purity. Statistical analysis was performed using the two-way analysis of variance (ANOVA) with a Tuckey Kramer test (n=30; 9 males); samples not connected by the same letter are significantly different ($p<0.05$). The data are presented as the mean ± the standard error of measurement (SEM). The y axis shows the response for sweetness on LMS scale, and the x axis is labeled with different water samples.

Additional details on human sensory triangle tests

Participants tasted each solution from individual plastic syringes and the procedure was repeated twice, without (‘taste and smell’ Figure S3A) and with (‘taste only’, Figure S3B) nose clips. The ‘taste only’ experiment included 26 participants (5 males) and the ‘smell and taste’ experiment included 28 subjects (9 males). In examining the effect of smell (Figure S3C), the experiment included 25 subjects (6 males) who were asked to smell each sample in a glass jar(5ml). For the data analysis, the total number of responses correctly identifying the ‘odd’ sample was counted and compared to a statistical table which determines the critical number (minimum) of correct answers required for a significant difference.

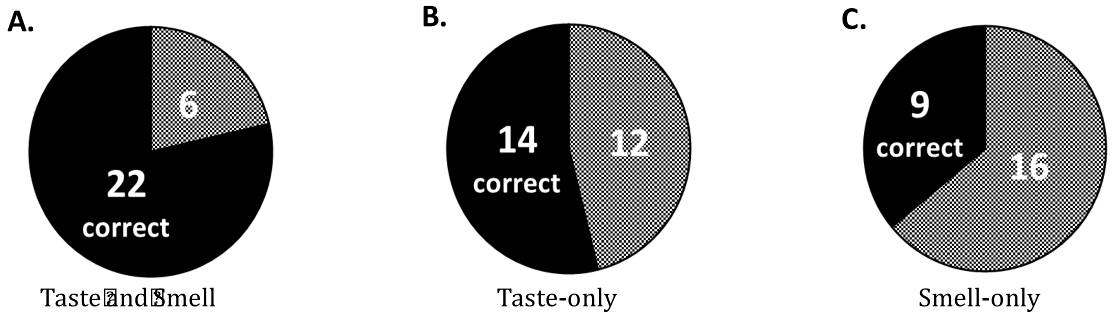

Figure S3. Discrimination tests of D_2O. D_2O can be distinguished from H_2O based on taste. Numbers within a circle indicate the number of subjects that correctly (black) or incorrectly (grey) identified the odd solution. Significant differences were observed in the case of open nose taste test (A; $p=0.001$) and in taste only test (B; $p=0.03$) using statistical table which determines the critical number (minimum) of correct answers required for a significant difference.

Cyclamate sweetness in two types of water after exclusion of two outliers

D_2O was perceived, on average, as “slightly sweet” and typically added to the sweetness of low concentrations of known sweeteners (D-glucose, sucrose and cyclamate), as shown in Fig 2. We noticed that 2 out of 30 subjects experienced reduction of sweetness perception of the moderate concentrations of cyclamate in heavy water compared to lower concentrations. Figure S4 shows average results excluding these individual cases, which leads to significantly higher sweetness of 4.5mM cyclamate in D_2O compared to H_2O.

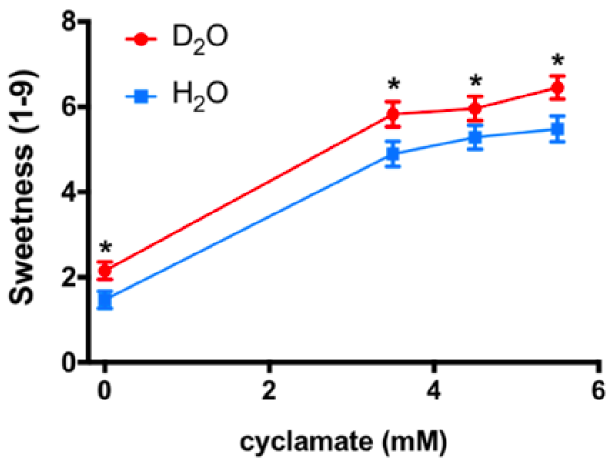

Figure S4. The effect of D_2O (red) compared to H_2O (blue) on cyclamate. Asterisks indicate a significant ($p < 0.05$) difference between water types using the two-way analysis of variance (ANOVA) with a pre-planned comparison t-test. All data are presented as the mean ± the Standard Error of Measurement (SEM); n=28 (8 males). The y axis shows the response for sweetness, while the x axis is labeled with different concentrations. Scale for sweetness is labeled as 1 = no sensation, 3 = slight, 5 = moderate, 7 = very much, and 9 = extreme sensation.

Additional details concerning experiments on mice

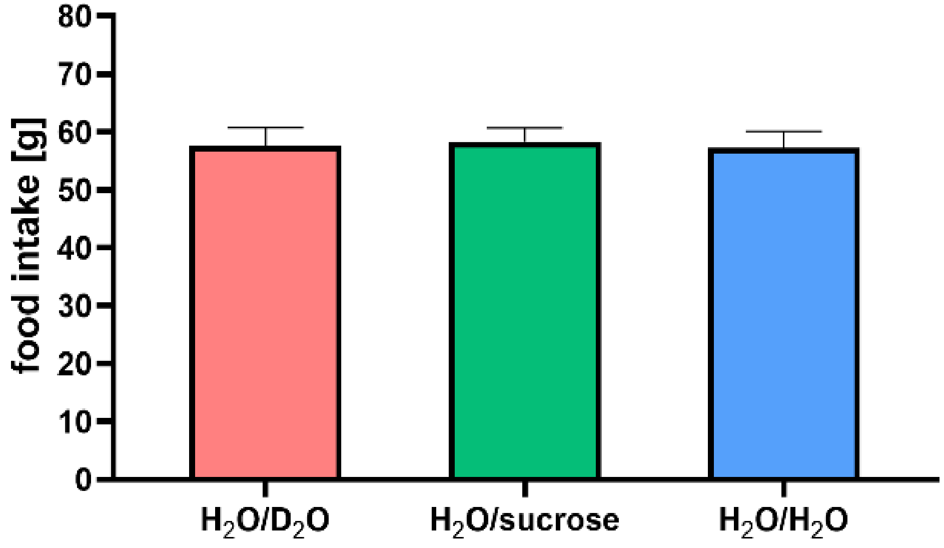

Figure S5. Food intake during experiment. Mice were placed in groups of two in cages. The data are presented as the mean ± standard error of measurement (SEM). Statistical analysis was performed using the one-way ANOVA with a Dunnett's test. (n = 10-12)

Table S1. Design of the animal experiment.

condition 1	condition 2	control
H_2O	H_2O	H_2O
D_2O	sucrose	H_2O

Homology modeling and sequence comparison

Several models of the transmembrane region of TAS1R3 and Tas1r3 were predicted by I-TASSER server. The best predicted model was chosen for further analysis, according to C-scores. mGluR1 and mGluR5 structures (PDB IDs: 4OR2 and 4OO9, respectively) were used as templates for constructing the models. Selected human and mouse models were then minimized and refined, using scwrl4(*2*) and Schrodinger program suits (version 11.2, Schrodinger, LLC, New York, NY, 2014).

Positions of H_2O molecules were compared among mGluR5 structures (4OO9, 5CGC, and 5CGD) and two conserved positions were found in the area of the TMD of mGluR5 structures.

Docking was performed for cyclamate and lactisole, to both human and mouse TAS1R3, in order to elucidate putative interaction of these compounds on the allosteric binding active site of the TMD of TAS1R3 and Tas1r3. Both ligands could be docked at the allosteric pocket of TAS1R3, but neither cyclamate nor lactisole docked into Tas1r3 model. This is in accord with experimental data showing that mice are not sensitive to cyclamate and lactisole.

Development of effective D_2O model for simulations

We have developed a new heavy water model SPC/E-HW (for intermolecular force field parameters of all the employed water models see Table S2) which effectively takes into account nuclear quantum effects (zero point motions in particular) by modifying the interaction potential for classical molecular dynamics. We were forced to develop a new model since the existing SPC/HW model (based on the same principles)(*3*) is not reproducing experimental properties of D_2O very well (see comparison with the present model in Tables S3 and S4). The results presented in Tables S3 and S4 were obtained using MD simulations with a time step of 2 fs, a long-range interaction cut-off of 1.2 nm, long range corrections for energy and pressure, the Nose-Hoover thermostat(*4*) with a coupling constant of 1.0 ps, and the Parrinello-Rahman barostat(*5*) with a coupling constant of 2.5 ps and a compressibility of $5 \cdot 10^{-5}$ bar^{-1}. The viscosity of the water model was calculated by the Green-Kubo formula(*6*). Our newly developed SPC/E-HW model underestimates the experimental viscosity of heavy water by ~20 % (Table S3). This is a significant improvement from the previous SPC/HW model that has an error in viscosity of almost 30 % (Table S3). Moreover, our heavy water model errs in viscosity similarly to the standard SPC/E model for light water. Indeed, the widely used SPC/E underestimates the viscosity of water by ~18 %. These errors in viscosity are not expected to affect the reported results, such as radii of gyration of proteins and relative sizes of root mean square fluctuations of individual amino acids. These are thermodynamic quantities and, as such, do not depend on the kinetic properties of the system, such as viscosity. All simulations were performed using the Gromacs program package(*7*), version 5.1.2.

Table S2: Oxygen Lennard-Jones parameters σ and ε and charges q_O of the water models used in this paper. All of them use the SPC/E geometry where the distance between oxygen and hydrogen atoms (d_{OH}) is 1 and the angle between those atoms of 109.47°.

Water model	σ/nm	ε/(kJ/mol)	q_O/e^-
SPC/E-HW	0.31970	0.5050	0.8376
SPC/HW(*3*)	0.31657	0.6497	0.8700
SPC/E(*8*)	0.31656	0.6502	0.8476

Table S3: Densities, temperatures of maximum density (TMD), and viscosities of the old (SPC/HW) and present (SPC/E-HW) heavy water models and the standard SPC/E normal water model. The experimental values are given in brackets.

	Density $\rho/(kg^3/m)$	TMD/K	Viscosity η/(mPa·s)
SPC/E-HW	1104.0±0.3<br>(1104.4)	250±1 (284.3)	0.88<br>(1.097)
SPC/HW(*3*)	1125.6±0.3<br>(1104.4)	268±1 (284.3)	1.40<br>(1.097)
SPC/E(*8*)	999.2±0.4<br>(997.1)	248±1 (277.1)	0.73<br>(0.890)

Table S4: Density differences, temperature of maximum density (TMD) differences, and viscosity differences between heavy and light water for the old (SPC/HW) and present (SPC/E-HW) heavy water models (with the standard SPC/E normal water model as a reference). Experimental values are in brackets.

	$\Delta\rho/(kg/^3m)$	ΔTMD/K	$\delta\eta$(%)
SPC/HW(*3*)	126.4<br>(107.3)	20<br>(7.2)	91.8<br>(23.3)
SPC/E-HW	104.8<br>(107.3)	2<br>(7.2)	20.6<br>(23.3)

Molecular dynamics simulations

The initial structure of the trans-membrane part of the human sweet taste receptor was designed by the homology modeling described above. It was then embedded in a membrane bilayer formed of 128 POPC lipids solvated either in H_2O or D_2O, with chloride added to neutralize the systems, employing the CHARMM36 forcefield(*9*). For each water model a total of 5 µs has been run at 298K and 1 atm, with about 3 µs used for analysis. All simulations were performed using the Gromacs program package(*7*), version 5.1.2.

We checked robustness of the MD simulations for the TMD of TAS1R3 by generating three independent trajectories and plotting the observables for each one separately. As seen from Figures S6 (and Figure 7B in the main paper), our sampling is sufficient and both differences in root mean square fluctuations and the radii of gyration are reasonably converged. Figure S7 displays for three independent simulation runs the time evolutions of the root mean square deviations, from which the RMSF and differences thereof in H_2O vs D_2O were evaluated.

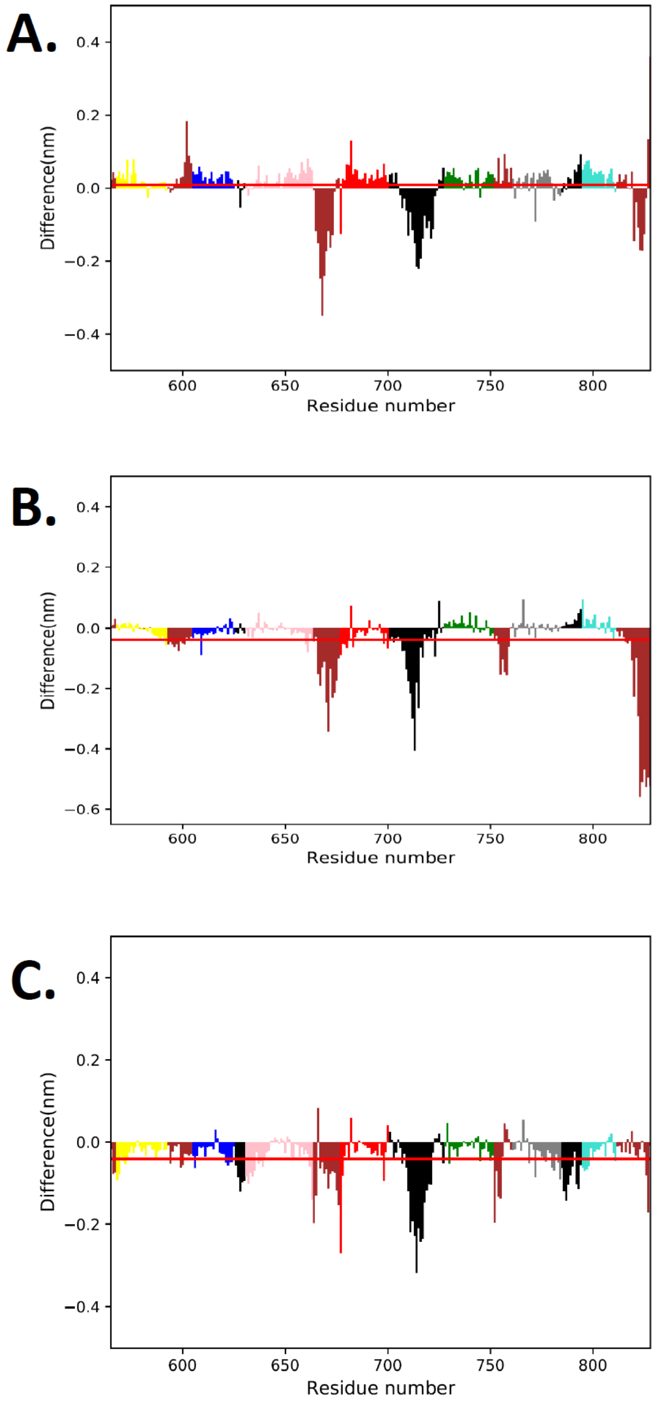

Figure S6: Differences in root mean square fluctuations of individual residues and their sum (red) for three separate microsecond-timescale simulations (color-coding is the same as in Figure 7D in the main paper).

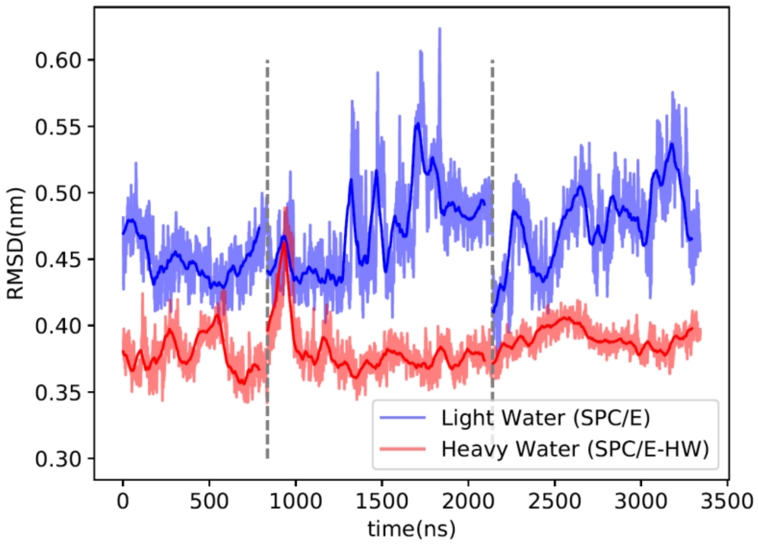

Figure S7: Time evolution of the RMSD in H_2O vs D_2O for three independent microsecond-timescale simulations (separated by vertical dashed lines).

In addition, we performed MD simulations of two other proteins - apo-azurin (PDB: 1E65) and ribonuclease-T1 (PDB:2RNT) – for which experimental data in H_2O and D_2O were available and the effect of heavy water on structural flexibility was studied(*10*). We employed the CHARMM36 forcefield(*9*). The proteins were solvated and Na^+ ions were added to zero the total charge. This system was energy-minimized and then simulations (two replicas) were run for 700 ns at 298K and 1 atm using the Nose-Hoover thermostat(*4*) and the Parrinello-Rahman barostat(*5*). For the time averaged analysis, the first 200 ns were discarded. We checked that the root mean square displacements from the initial structure did not drift in time and were relatively small (< 2 Å). All simulations were performed using the Gromacs program package(*7*), version 5.1.2.

From the MD simulations of the proteins in H_2O (SPC/E[52]) and D_2O (SPC/E-HW, see below) we calculated the time evolution of the radii of gyration. We see that most residues are slightly more rigid and the protein structures are a slightly more compact in

D_2O compared to H_2O (Figure S8), which is consistent with the experimentally observed higher protein rigidity in the former solution(*10*).

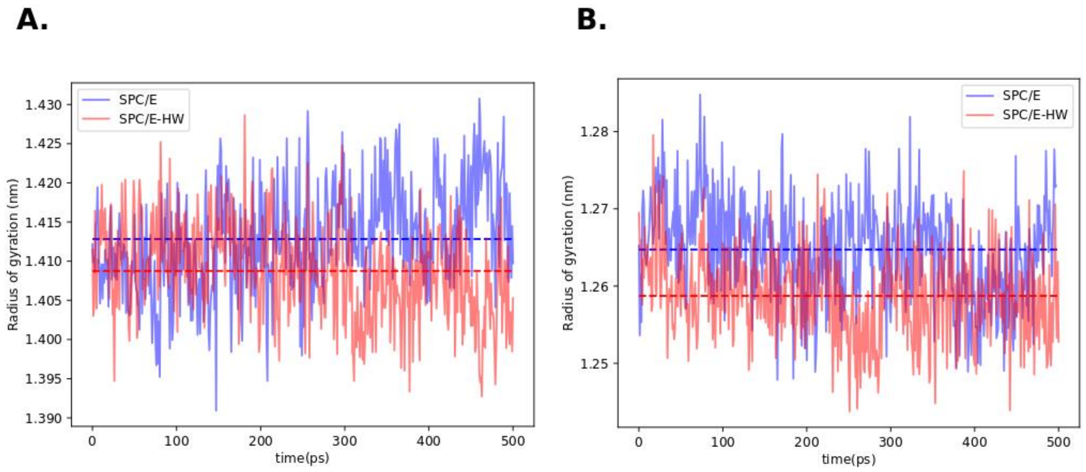

Figure S8: Time evolutions of the radii of gyration in H_2O (blue) and D_2O (red) with mean values as dashed lines, showing that both (a) apo-azurin and (b) ribonuclease-T1 are more compact in heavy water.

References

1. B. G. Green, G. S. Shaffer, M. M. Gilmore, Derivation and Evaluation of a Semantic Scale of Oral Sensation Magnitude with Apparent Ratio Properties. *Chem Senses* **18**, 683-702 (1993).
2. G. G. G. G. Krivov, M. V. Shapovalov, J. R. L. Dunbrack, R.L. , Improved Prediction of Protein Side-Chain Conformations With SCWRL4 *Proteins* **77**, 778-795 (2009).
3. J. R. Grigera, An effective pair potential for heavy water. *Journal of Chemical Physics* **114**, 8064-8067 (2001).
4. W. G. Hoover, Canonical dynamics: Equilibrium phase-space distributions. *Physical Review A* **31**, 1695-1697 (1985).
5. M. Parrinello, A. Rahman, Polymorphic transitions in single crystals: A new molecular dynamics method. *Journal of Applied Physics* **52**, 7182-7190 (1981).
6. B. Hess, Determining the shear viscosity of model liquids from molecular dynamics simulations. *The Journal of Chemical Physics* **116**, 209-217 (2002).
7. S. Pronk *et al.*, GROMACS 4.5: a high-throughput and highly parallel open source molecular simulation toolkit. *Bioinformatics* **29**, 845-854 (2013).
8. H. J. C. Berendsen, J. R. Grigera, T. P. Straatsma, The Missing Term in Effective Pair Potentials. *Journal of Physical Chemistry* **91**, 6269-6271 (1987).
9. J. Huang *et al.*, CHARMM36m: an improved force field for folded and intrinsically disordered proteins. *Nature Methods* **14**, 71-73 (2017).
10. P. Cioni, G. B. Strambini, Effect of heavy water on protein flexibility. *Biophysical journal* **82**, 3246-3253 (2002).
