# Supplementary material for: Sweet taste of heavy water
Source: Commun Biol. 2021 Apr 6;4:440. doi: 10.1038/s42003-021-01964-y (PMC8024362; doi:10.1038/s42003-021-01964-y)
Supplement: Supplementary file 2 — Description of Supplementary Files [file 42003_2021_1964_MOESM2_ESM.pdf]

Description of Additional Supplementary Files

File name: Supplementary Data 1
Description: Raw data for Figure 2.

File name: Supplementary Data 2
Description: Raw data for Figure 3.

File name: Supplementary Data 3
Description: Raw data for Figure 4.

File name: Supplementary Data 4
Description: Raw data for Figure 5a.

File name: Supplementary Data 5
Description: Raw data for Figure 5c.

File name: Supplementary Data 6
Description: Raw data for Figure 6.

File name: Supplementary Data 7
Description: Raw data for Figure 7b.

File name: Supplementary Data 8
Description: Raw data for Figure 7d.
