# Supplementary material for: Sweet taste of heavy water
Source: Commun Biol. 2021 Apr 6;4:440. doi: 10.1038/s42003-021-01964-y (PMC8024362; doi:10.1038/s42003-021-01964-y)
Supplement: Supplementary file 11 — Reporting Summary [file 42003_2021_1964_MOESM11_ESM.pdf]

nature research

Corresponding author(s): Pavel Jungwirth

Last updated by author(s): Dec 8, 2020

Reporting Summary

Nature Research wishes to improve the reproducibility of the work that we publish. This form provides structure for consistency and transparency in reporting. For further information on Nature Research policies, see our Editorial Policies and the Editorial Policy Checklist.

Statistics

For all statistical analyses, confirm that the following items are present in the figure legend, table legend, main text, or Methods section.

n/a	Confirmed	
☐	☒	The exact sample size (*n*) for each experimental group/condition, given as a discrete number and unit of measurement
☐	☒	A statement on whether measurements were taken from distinct samples or whether the same sample was measured repeatedly
☐	☒	The statistical test(s) used AND whether they are one- or two-sided<br>*Only common tests should be described solely by name; describe more complex techniques in the Methods section.*
☐	☐	A description of all covariates tested
☐	☒	A description of any assumptions or corrections, such as tests of normality and adjustment for multiple comparisons
☐	☒	A full description of the statistical parameters including central tendency (e.g. means) or other basic estimates (e.g. regression coefficient) AND variation (e.g. standard deviation) or associated estimates of uncertainty (e.g. confidence intervals)
☐	☒	For null hypothesis testing, the test statistic (e.g. F, t, r) with confidence intervals, effect sizes, degrees of freedom and P value noted<br>*Give P values as exact values whenever suitable.*
☒	☐	For Bayesian analysis, information on the choice of priors and Markov chain Monte Carlo settings
☒	☐	For hierarchical and complex designs, identification of the appropriate level for tests and full reporting of outcomes
☒	☐	Estimates of effect sizes (e.g. Cohen's *d*, Pearson's *r*), indicating how they were calculated

Our web collection on statistics for biologists contains articles on many of the points above.

Software and code

Policy information about availability of computer code

Data collection	Automatic drinking monitoring system (Developmental Workshops of Institute of Organic Chemistry and Biochemistry of the Czech Academy of Sciences, Prague, Czech Republic)
Data analysis	GraphPad Prism 8 software (GraphPad Software, Inc., San Diego, CA, USA)

For manuscripts utilizing custom algorithms or software that are central to the research but not yet described in published literature, software must be made available to editors and reviewers. We strongly encourage code deposition in a community repository (e.g. GitHub). See the Nature Research guidelines for submitting code & software for further information.

Data

Policy information about availability of data

All manuscripts must include a data availability statement. This statement should provide the following information, where applicable:

- Accession codes, unique identifiers, or web links for publicly available datasets
- A list of figures that have associated raw data
- A description of any restrictions on data availability

The datasets generated during the current study are available from the corresponding author on reasonable request.

Field-specific reporting

Life sciences study design

All studies must disclose on these points even when the disclosure is negative.

Sample size	based on ISO 8586:2012, it is highly recommended that a panel has at least 10 selected assessors.
Data exclusions	Exclusion criteria were heavy smoking, pregnancy, impaired sense of taste and food allergies disorders; the information was self-report based. Two participants were excluded from the final analysis in Cyclamate and Sucrose experiments due to an unusual grading of solutions (to decrease variation among tasters).
Replication	at least 3 replications for cell and human experiments (min. 15 subjects)
Randomization	Human experiments: taste samples were presented to the panelists in a randomized order using Cloud Compusense software
Blinding	Human experiments; investigators were blinded to identity of the panelist (source of data) during data collection and analysis

Reporting for specific materials, systems and methods

We require information from authors about some types of materials, experimental systems and methods used in many studies. Here, indicate whether each material, system or method listed is relevant to your study. If you are not sure if a list item applies to your research, read the appropriate section before selecting a response.

Materials & experimental systems

n/a	Involved in the study	
☒	☐	Antibodies
☐	☒	Eukaryotic cell lines
☒	☐	Palaeontology and archaeology
☐	☒	Animals and other organisms
☐	☒	Human research participants
☒	☐	Clinical data
☒	☐	Dual use research of concern

Methods

n/a	Involved in the study	
☒	☐	ChIP-seq
☒	☐	Flow cytometry
☒	☐	MRI-based neuroimaging

Eukaryotic cell lines

Policy information about cell lines

Cell line source(s)	HEK 293T cells from ATCC
Authentication	not authenticated
Mycoplasma contamination	cells were negative for mycoplasma
Commonly misidentified lines (See ICLAC register)	none

Animals and other organisms

Policy information about studies involving animals; ARRIVE guidelines recommended for reporting animal research

Laboratory animals	Mice, C57BL/6J, males, three months (Charles Rivers Laboratories, Sulzfeld, Germany)
Wild animals	The study did not involve wild animals
Field-collected samples	Mice were housed at a temperature of 23 °C and a daily cycle of 12 h light and dark (lights on at 6 am). The mice were placed in groups of two in cages in automatic drinking system, n = 34
Ethics oversight	All animal experiments followed the ethical guidelines for animal experiments and the Act of the Czech Republic Nr. 246/1992 and were approved by the Committee for Experiments with Laboratory Animals of the Czech Academy of Sciences

Note that full information on the approval of the study protocol must also be provided in the manuscript.

Human research participants

Policy information about studies involving human research participants

Population characteristics	Subjects between the ages 20 - 43 years. The study included 10 human experiments with different groups of participants:

	15-30 subjects each (between 4 to 12 males)
Recruitment	on-line advertisements
Ethics oversight	Committee for the Use of Human Subjects in Research in The Robert H. Smith Faculty of Agriculture Food and Environment, the Hebrew University of Jerusalem

Note that full information on the approval of the study protocol must also be provided in the manuscript.
